# The Two-Sided Experimental Model of ImmunoCAP Inhibition Test as a Useful Tool for the Examination of Allergens Cross-Reactivity on the Example of α-Gal and Mammalian Meat Sensitization—A Preliminary Study

**DOI:** 10.3390/cimb45020077

**Published:** 2023-02-01

**Authors:** Kinga Lis, Natalia Ukleja-Sokołowska, Kornelia Karwowska, Joanna Wernik, Małgorzata Pawłowska, Zbigniew Bartuzi

**Affiliations:** 1 Department of Allergology, Clinical Immunology and Internal Medicine, Ludwik Rydygier Collegium Medicum in Bydgoszcz, Nicolaus Copernicus University in Toruń, ul. Ujejskiego 75, 85168 Bydgoszcz, Poland; 2 Department of Infectious Diseases and Hepatology, Ludwik Rydygier Collegium Medicum in Bydgoszcz, Nicolaus Copernicus University in Toruń, ul. Świętego Floriana 12, 85030 Bydgoszcz, Poland

**Keywords:** cross-reactivity, inhibition test, ImmunoCAP, α-GAL, mammalian meat allergens

## Abstract

Cross-reactivity of allergens is the cause of various, sometimes unexpected, clinical reactions. There are no standard methods to investigate cross-reactivity. We present an experimental model of a two-sided inhibition test (IT) on ImmunoCAP membranes (CAP). We constructed the described model based on the known cross-allergy syndrome to red meat developing in people bitten by ticks (α-Gal syndrome; AGS). Some individuals who are bitten by ticks develop IgE antibodies specific to the carbohydrate determinant, galactose-α-1,3-galactose (α-Gal), present in the tick’s saliva. These antibodies can cross-react with α-Gal molecules expressed on mammalian meat proteins. The well-known property of anti-α-Gal IgE antibodies binding by various sources of this allergen was used by us in the proposed model of the two-sided inhibition test on ImmunoCAP membranes. We expected that anti-α-Gal IgE antibodies bind allergens from mammalian meat and blocking them abolishes this reactivity, and the two-sided inhibition test model we proposed on ImmunoCAP membranes allowed us to observe such a relationship. We conducted the experiment three times on biological material from people with different clinical manifestations of allergy to α-Gal, each time obtaining similar results. In conclusion, the model of bilateral inhibition on ImmunoCAP membranes proposed by us seems to be an attractive, simple tool for direct testing of allergic cross-reactivity.

## 1. Introduction

The cross-reactivity of allergens is a result of the similar structure of their antigenic determinants, both their linear and spatial structure [1]. It may also be related to the presence of cross-reacting carbohydrate determinants [2]. We expect that the more similar the structure of antigens is, the more often there will be cross-linking of antibodies specific for allergens from different sources. However, the observed clinical cross-reactions are not always as expected. This may be related to several factors, including changes that affect proteins during post-translational processing, the unique microenvironment of blood serum and the polyclonal nature of naturally occurring antibodies. Various types of inhibition tests are used in the study of the cross-reactivity of allergen-specific antibodies in conditions similar to natural. They are successfully used in various experimental systems [3,4,5,6,7,8,9,10,11,12], both with the use of natural allergen extracts and their standardized solutions. Although the results of these experiments are very promising, they are still not standardized. Therefore, it seems necessary to undertake further research in this area to obtaining an effective and reliable tool for the assessment and prediction of the clinical manifestation of sensitization to allergens with the expected potential for cross-reactivity.

Alpha-gal (galactose-α-1,3-galactose, α-Gal) is a carbohydrate present on glycoproteins in non-primate mammals and certain invertebrates. Primates, including humans, do not express the α-Gal epitope, as the -1,3-galactosyltransferase gene is inactivated in these species [1,2,3,4]. A lack of α-Gal expression results in the production of different classes of antibodies (IgM/IgG/IgE) targeting these carbohydrate determinants. α-Gal is transferred to the human body with the tick’s saliva during the bite. In some individuals, this stimulates the immune system to produce α-Gal-specific IgE. This may later result mild-to-severe allergic reactions to mammalian meat (as beef, pork, lamb, or other) [13,14,15]. This unusual allergic reaction, associated with an allergy to α-Gal, is called α-Gal syndrome (AGS) [16,17].

AGS can manifest as an immediate drug allergy to pharmaceuticals containing α-Gal and/or a delayed hypersensitivity response to the ingestion of mammalian meat products. The first references to an association between alpha-gal allergy and red meat allergy appeared around 2006 and concerned unusual allergic reactions in people treated with cetuximab. Later, it was noticed that people who had been bitten by ticks previously manifested similar symptoms after ingestion of the mammalian meat. In all cases, the common factor was the presence of IgE antibodies to the carbohydrate determinant galactose-α-1,3-galactose, referred to as α-Gal [16,17].

Although the mechanism for the huge variability of the clinical symptoms of AGS and the delay in response is not entirely clear, it is known that IgE antibodies specific for α-Gal resulting from primary sensitization to tick saliva may bind this carbohydrate determinant expressed on mammalian meat proteins [18,19,20,21,22]. It is also unclear why not all types of mammalian meat cause AGS symptoms equally, and beef and beef offal appear to be the strongest stimulants of these responses [23].

The aim of the study was to evaluate the usefulness of the experimental two-sided inhibition test (IT) on ImmunoCAP membranes for the study of cross-reactivity of allergens. In order to achieve the assumed aim of the work, an exemplary model based on the known reactivity of α-Gal specific IgE antibodies with mammalian meat allergens was used.

## 2. Materials and Methods

Two-side ImmunoCAP inhibition tests with two allergens (α-Gal and beef) were performed to assess the cross-reactivity between α-Gal antigen and mammalian meat allergens.

The study was carried out by case-to-case analysis. The serum of three patients with positive results anti-α-Gal IgE and anti-beef IgE, negative results anti-poultry IgE and different clinical histories associated with meat consumption and different underlying atopic status was used in the experimental inhibition assay.

### 2.1. Patients History

Patient 1—A 65-year-old man was diagnosed with recurrent anaphylactic reactions, which he initially associated with the consumption of various types of alcohol. Clinical history revealed that alcohol consumption was always accompanied by a meal consisting of a meat dish. In addition, episodes of anaphylaxis also occurred in association with exercise immediately before or immediately after eating a meat meal. The first episode of anaphylaxis occurred about 9 years ago. The man showed no allergic reactions before this event. It was established that about a year before the first episode of anaphylaxis, the man was bitten by a tick. The species of the tick could not be determined. The reason for the determination of anti-α-Gal IgE antibodies in this patient was clinical symptoms related to meat consumption and the presence of specific IgE antibodies to mammalian meat allergens detected in the ALEX2 multiplex test.

Patient 2—A 60-year-old man was treated at an infectious disease clinic due to skin erythema associated with a tick bite. The bite occurred 2 months earlier. In addition to a diagnosis appropriate to the clinical situation, the man was tested for the presence of anti- α-Gal IgE antibodies as part of another clinical study conducted by our team, which aimed to determine the frequency of these antibodies in people bitten by ticks. The man denied any symptoms of allergies, including meat allergies. He ate the meat regularly and had no negative effects.

Patient 3—A 40-year-old woman diagnosed with oral allergy syndrome (OAS) symptoms after consuming a variety of fruits, vegetables and nuts, including sweet cherries, nectarines, cherries, apples, hazelnuts and almonds. The patient had previously been diagnosed with bronchial asthma and inhalation allergy to hazel, birch, grass, weeds, Alternaria, mites, cats and dogs. In the course of the conducted diagnostics, the patient was found to have IgE specific for mammalian meat allergens. The patient denies the symptoms of an allergy to meat, which she consumes regularly. The woman was not sure if she had ever been bitten by a tick. The patient was tested for IgE antibodies to alpha-gal due to the positive IgE results of mammalian meat allergens in the ALEX2 test.

### 2.2. Laboratory Procedures

The following laboratory tests were performed in the blood serum collected from each patient: the concentration of specific IgE antibodies to α-Gal (ImmunoCAP, Thermo Fisher Scientific, Waltham, MA, USA), wide-profile multiplex tests with the ALEX2 (Allergy Xplorer, MacroArray Diagnostics, Vienna, Austria; MADx) and ISAC*_E112i_* (ImmunoCAP, Thermo Fisher Scientific, Waltham, MA, USA) tests, and the concentration of IgE specific for mammalian meat allergens (beef, pork, rabbit meat, mutton), gelatin and chicken meat (ImmunoCAP, Thermo Fisher Scientific, Waltham, MA, USA).

In the sera, two-side inhibition tests were performed on two types ImmunoCAP matrices: o215 (alpha-gal, bovine thyroglobulin; TBG) and f27 (beef meat extract). To assess the effectiveness of the inhibition test and the cross-reactivity between α-Gal and meat allergens, the following tests were performed in the material collected after the inhibition tests: specific IgE concentration for α-Gal and beef (ImmunoCAP) and the multiplex ALEX2 test.

Each patient’s serum was processed separately. Serum pooling was not performed before or after the inhibition test.

A standard, certified for in vitro diagnostic (CE IVD), laboratory methods (ImmunCAP, ISAC*_E112i_* test, ALEX2 test), and experimental two-side inhibition tests on ImmunoCAP matrices were used in the experiment.

#### 2.2.1. Standard Laboratory Methods

The ImmunoCAP (Thermo Fisher Scientific, Waltham, MA, USA) is the in vitro test system for the quantitative measurement of specific IgE in human serum or plasma. In the ImmunoCAP system, the FEIA (fluoro-immuno-enzymatic) method is use. The allergens covalently coupled to ImmunoCAP, reacts with the specific IgE from the patient sample. After washing non-specific particles enzymes labeled anti-IgE (mouse monoclonal β-galactosidase-anti-IgE) are added to form the complex. Following incubation, unbound conjugate (enzyme-anti-IgE) is washed away, and the bound complex is then incubated with a developing agent (4-methylumbelliferyl-β-D-galactoside). After stopping the reaction (by sodium carbonate 4%), the fluorescence of the eluate is measured. The higher the response value, the more specific IgE is present in the sample. To evaluate the test results, the responses for the patient samples are transformed to concentration with the use of a calibration curve, with a calibrator range of 0–100 kUA/L. The Phadia 100 system was used in the experiment.

The ISAC*_E112i_*test (Thermo Fisher Scientific, Waltham, MA, USA) is an enzyme immunoassay solid phase based on biochip (microarray) technology. Allergen components applied in triplets to a glass plate (slide) bind their specific IgE antibodies from the patient’s blood serum. The specific IgE bound in this way is then detected with fluorescently labeled anti-human immunoglobulin E (anti-human IgE) antibodies. Fluorescence is measured with a biochip scanner (CapitalBio LuxScan 10k, CapitalBio, Hong Kong, China). The obtained images are then analyzed using Phadia Microarray Image (MIA) software. The ISAC*_E112i_* is a semi-quantitative test, and specific IgE antibody levels are reported in ISAC Standardized Units (ISU-E) and are divided into four categories according to the level: <0.3 ISU-E (undetectable); 0.3–0.9 ISU-E (low); 1–14.9 ISU-E (medium/high); >15 ISU-E (very high). The CapitalBIO LuxSCAN system with PhadiaMIA software was used in the experiment.

The ALEX2 (Allergy Xplorer, MacroArray Diagnostics; MADx) is an immunological, microarray test based on nanotechnology. The ALEX2 enables the simultaneous measurement of the concentration of specific IgE for whole allergen extracts, allergen molecules and total immunoglobulin E. The ALEX2 is a quantitative specific IgE and semi-quantitative total IgE test. The measured immunoglobulin concentration is expressed in kUA/L for specific IgE and kU/L for total IgE. The measuring range of the ALEX2 test for specific IgE is 0.3–50 kUA/L and for total IgE 1–2500 kU/L. The sensitivity of the test for specific IgE is 0.1 kUA/L. The results for specific IgE were expressed in classes 0–4, where individual classes were assigned the concentration of specific IgE, respectively: class 0 (<0.3 kUA/L; negative or borderline result), class 1 (0.3–1 kUA/L; concentration low), class 2 (1–5 kUA/L; medium concentration), class 3 (5–15 kUA/L; high concentration) and class 4 (>15 kUA/L; very high concentration). The manual Image Xplorer system with Raptor software was used in the experiment.

Singleplex and multiplex results were analyzed separately.

#### 2.2.2. ImmunoCAP Inhibition Test (IT)

We designed a three-step experimental procedure to evaluate the cross-reactivity between α-Gal and different mammalian meat allergens (beef, pork, rabbit, horse meat, sheep meat).

As a source of allergens, we used two types of ready-made ImmunoCAP membranes: coated with α-Gal (o215) and coated with beef extract (f27) (Thermo Fisher Scientific, Waltham, MA, USA).

The source of specific IgE antibodies were sera of patients with positive specific IgE antibodies to α-Gal (necessary condition) and beef (necessary condition) and various allergens of mammalian meat (optional condition) and negative specific IgE antibodies to poultry meat allergens (necessary condition).

**Figure 1:**
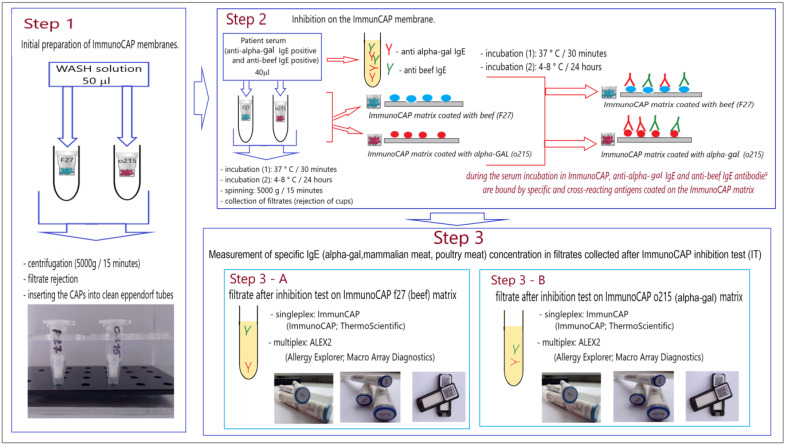
ImmunoCAP inhibition test with o215 (alpha-gal, ImmunoCAP) and f27 (beef, ImmunoCAP)—the experiment scheme.

The scheme of the experiment is presented in Figure 1.

#### 2.2.3. The Experiment Scheme

The elements of the ImmunoCAP system (Thermo Fisher Scientific, Waltham, MA, USA) were used in the experiment. The ImmunoCAP method is a standard method for the determination of serum specific IgE or total IgE concentration. The ImmunoCAP membrane is a cellulose matrix that is the solid phase in the ImmunoCAP method. The membrane placed in the plastic reaction cup is the CAP of the ImmunoCAP system. The ImmunoCAP membrane is coated with allergens or anti-human IgE antibodies. In the standard ImmunoCAP procedure, it binds antibodies to the serum.

*Step 1*

This stage was aimed at preparing immunocap membranes for binding IgE antibodies from patients with membrane allergens (solid phase), as in the standard ImmunoCAP procedure.

In the first stage of the experiment, the ImmunoCAP membranes were pre-prepared. For this purpose, ImmunoCAPs (CAPs) were placed in Eppendorf tubes and 50 µL of the washing solution was added to each CAP. The washing buffer used in this step of the experiment is part of the ImmunoCAP system (Art. No. 10-9422-01). The buffer was prepared in accordance with the manufacturer’s instructions. The CAPs sealed in Eppendorf tubes were then centrifuged (5000× *g*; 15 min) to remove the wash buffer from the CAPs. The CAPs prepared in this way were placed in clean Eppendorf tubes.

*Step 2*

In this stage of the experiment, we assumed that the anti-α-Gal IgE and anti-beef IgE antibodies present in the sera and other anti-mammalian meat IgE(optional) would be inhibited by α-Gal and/or beef antigens coated on the ImmunoCAP membranes. For this purpose, 40 µL of anti-α-Gal IgE and anti-beef IgE positive sera and anti-mammalian meat (optional) were applied to the pre-prepared ImmunoCAP membranes. ImmunoCAP membranes with applied sera were tightly sealed in tubes and subjected to two-stage incubation: first 30 min at 37 °C and then 24 h in a refrigerator (2–4 °C). After incubation, the tubes containing the cups of ImmunoCAP were centrifuged (5000× *g*; 15 min). After completion of centrifugation, the ImmunoCAP membranes were removed, and the collected filtrate was used in the next stage of the experiment (Step 3).

Two types of filtrate were obtained for each sample: (A) the filtrate after the inhibition test on the ImmunoCAP f27 (beef; f27-ImmunoCAP inhibition test; f27-IT) and (B) the filtrate after the inhibition test on the ImmunoCAP o215 (α-Gal; o215-ImmunoCAP inhibition test; o215-IT).

Because the volume of serum required for the third stage of the experiment was 210 µL (for each patient), 6 CAPs of ImmunoCAP o215 (α-Gal coated membrane) and f27 (beef-coated membrane) were prepared for each patient. A 240 µL volume of the final serum of each patient was obtained.

*Step 3*

In this stage of the experiment, the effectiveness of the performed inhibition tests: f27-IT (Step 3 A) and o215-IT (Step 3 B) was verified. For this purpose, the concentration of IgE antibodies specific for beef and alpha-gal in the collected filtrates using the ImmunoCAP technique was determined, and the ALEX2 multiplex test was performed.

### 2.3. Control

In the presented experimental inhibition test, we focused on blocking antibodies to mammalian meat allergens and to alpha-Gal, which is the result of cross-reactivity. The test should have no significant effect on the concentration of IgE specific for other allergens. Because we used the multi-parameter ALEX2 test in the experiment, it allowed us to detect the presence of IgE antibodies specific for various allergens in the patients’ sera. In this way, we obtained an individual sensitization profile for each patient, which was used as a control for inhibition specificity. We compared the results of the ALEX2 test performed before and after the inhibition test, focusing on changes in IgE concentrations specific to allergens other than mammalian meat. The comparison was made individually for each patient. 

### 2.4. Statistical Analysis

Two non-parametric tests were used for the statistical analysis of the test results: the Wilcoxon test and the Friedman ANOVA test.

Only the results of inhibition tests for IgE antibodies to mammalian meat allergens and a-Gal were analyzed. The very small sample size limits the interpretation of the results of this analysis. 

No statistical analysis was performed for the controls due to the individual nature of these results.

## 3. Results

All results were divided into four groups: results before ImmunoCAP inhibition tests (IT), results after ImmunoCAP inhibition test with α-Gal (o215-ImmunoCAP inhibition test; o215-IT), results after ImmunoCAP inhibition test with beef (f27-ImmunoCAP inhibition test; f27-IT) and control results. All of them are presented in Table 1 and Table 2.

### 3.1. Results before ImmunoCAP Inhibition Test

The total serum concentration of IgE in each of the described patients was very high and, in each case, exceeded the value of 100 kU/L, which is considered the upper range of reference values for adults. The level of IgE was particularly high in Patient 1 (2602 kU/L) and Patient 2 (714 kU/L) (Table 1).

Anti-α-Gal (o215) IgE antibodies were found in the serum of every patient by the monospecific ImmunoCAP test. This was the first inclusion criterion for the experiment. The concentration of these antibodies in Patient 1 was extremely high and amounted to 302 kUA/L (quantification of the concentration required dilution of the serum). In two cases (Patient 1 and Patient 2) the presence of IgE antibodies against α-Gal was confirmed by multi-component ISAC*_E112i_*. Positive results as measured by the ImmunoCAP monospecific test were proportional to those measured by the ISAC test in both Patient 1 (302 kUA/L vs. 12 ISU-E) and Patient 2 (35.8 vs. 0.3 ISU-E). The concentration of IgE anti-α-Gal in the serum of Patient 3 measured by the ImmunoCAP test was very low (0.7 kUA/L), which was reflected in the negative result of the semi-quantitative ISAC*_E112i_* test for this parameter (<0.3 ISU-E) (Table 1).

The ratio of anti-α-Gal IgE to total serum IgE (anti-α-Gal IgE to total IgE ratio) between patients varied widely, being 11.61% in Patient 1, 5.01% in Patient 2 and only 0.52% in Patient 3 (Table 1).

The presence of anti-beef IgE was the second inclusion criterion for the study. IgE antibodies to beef allergens (ImmunoCAP: anti-f27; ALEX2: anti-Bos d_meat) were detected in the serum of all patients, but their concentration varied. Although these antibodies were detected in each of the three tested patients by both the ImmunoCAP monospecific test and the ALEX2 multiparameter quantitative test, the anti-beef IgE concentrations obtained in both tests were not mutually proportional. In Patient 1, the concentration of anti-beef IgE in the ImmunoCAP test was high (26 kUA/L), whereas in the ALEX2 test it was only 2.03 kUA/L. The inverse relationship occurred in Patient 2, where the concentration of IgE anti-beef antibodies in the ImmunCAP test was significantly lower than that measured with the ALEX2 test (0.38 kUA/L vs. 3.18 kUA/L). In turn, in Patient 3, both tests obtained similar results for this parameter (ImmunoCAP vs. ALEX2: 0.51 kUA/L vs. 0.53 kUA/L) (Table 1).

All patients were also tested for the presence of IgE antibodies to meat allergens of other mammals (i.e., pork, lamb, rabbit meat) with the single-parameter ImmunoCAP tests. The IgE antibodies to these types of meat were present only in the serum of Patient 1. None of the examined patients had IgE antibodies specific for porcine gelatin. No antibodies to chicken meat were detected in any of the sera tested by the ImmunoCAP test (Table 1).

The allergenic profile to mammalian meat allergens obtained in the ALEX2 multiparameter test basically corresponded to the allergenic profile to these allergens obtained in the ImmunoCAP single-parameter tests only in Patient 1. In this patient, the presence of IgE antibodies in beef, pork and rabbit meat was confirmed by the ALEX2 test results. The presence of IgE antibodies to the mutton (f88; Ovia_meat) has not been confirmed. Additionally, in Patient 1, the presence of horse meat IgE antibodies was detected with the ALEX2 test (Table 1). In Patient 2, the sensitization profile to mammalian meat allergens obtained with the ALEX2 test differed significantly from the results of the ImmunoCAP single-parameter tests. In the serum of this patient, the ALEX2 test detected IgE antibodies to beef, pork, horse, rabbit and sheep meat, whereas the ImmunoCAP tests were positive only for beef (Table 1). In Patient 3, in the ALEX2 test, only IgE antibodies for beef were detected, similar to the ImmunoCAP tests. The presence of IgE antibodies in horse meat was found (ALEX2) in the gray zone (equivocal), i.e., between 0.1 kUA/L and 0.3 kUA/L (Table 1).

No IgE antibodies specific for the main allergenic components of beef (Bos d 6) and pork (Sus d 1) were detected by ALEX2 in any of the patients (Table 1).

The IgE antibodies specific for poultry meat allergens (chicken, turkey) were not detected in the serum of any of the Patients (Table 1). This was the third inclusion criterion for the experiment.

### 3.2. Results after ImmunoCAPInhibition Test with α-Gal (o215-ImmunoCAP Inhibition Test; o215-IT)

The decrease in total IgE concentration after the o215 (α-Gal) ImmunoCAP inhibition test (o215-IT) occurred in the serum of each patient and ranged from 14.93% to 97.2% (Table 1).

The decrease in anti-α-Gal IgE antibody concentration from baseline was observed in the serum of each patient (85.56%, 88.04% and 100%, respectively) (Table 1).

The anti-α-Gal IgE to total IgE ratio for Patient 1 and Patient 2 was higher than before the o215 ImmunoCAP inhibition test (27.25% vs. 11.61% and 21.4% vs. 5.0%, respectively). However, in Patient 3, it was lower than the initial one (0% vs. 0.52%) (Table 1).

After blocking on an α-Gal(o215-IT)-coated ImmunoCAP membrane, a reduction in the concentration of IgE antibodies to beef allergens was observed in the mono-parameter test (ImmunoCAP) in each patient. The lower base concentration of anti-beef IgE antibodies corresponded to its higher percentage decrease after the o215-IT test (Table 1).

In the results of the ALEX2 test, no positive IgE antibodies were observed for beef allergens in all the tested sera after the o215-ImmunoCAP inhibition test (Table 1).

For the other mammalian meat allergens (pork, rabbit, horse meat, sheep meat), a significant or complete decrease in serum IgE levels was observed compared to the initially positive results of these antibodies in individual patients (Table 1).

Antibodies to poultry and bovine serum albumin (Bos d 6) and porcine serum albumin (Sus d1) remained negative after the o215-ImmunoCAP inhibition test (Table 1).

### 3.3. Results after ImmunoCAP Inhibition Test with Beef (f27-ImmunoCAP Inhibition Test; f27-IT)

A decrease in total IgE concentration after the ImmunoCAP f27 (beef) inhibition assay (f27-IT) was observed in the serum of each patient. It was lower than after the o215-IT test and ranged from 4.47% to 42.99% (Table 1).

After inhibition on a beef-coated ImmunoCAP membrane (f27-IT), a reduction in the concentration of IgE anti-α-Gal antibodies in the sera of all patients was observed. The reduction was lower than when inhibited on the ImmunoCAP membrane coated with α-Gal (o215-IT). The relationship between f27-IT vs. o215-IT was for Patient 1: 46.69% vs. 85.56% and Patient 2: 27.09% vs. 88.04%. For Patient 3, the efficacy of both inhibition tests was 100% (Table 1).

The anti-α-Gal IgE to total IgE ratio for Patient 1 and Patient 2 was similar to before the o215 ImmunoCAP inhibition test (10.85% vs. 11.61% and 5.46% vs. 5.0%, respectively). However, in Patient 3 it was lower than the initial one (0.07% vs. 0.52%) (Table 1).

After inhibition on a beef-coated ImmunoCAP membrane (f27-IT), an almost 100% reduction in IgE antibody concentration for bovine allergens was observed, which was measured by the mono-parameter test (ImmunoCAP). In Patient 2 and Patient 3, the concentration of the anti-beef IgE antibody in the blocked sera was below 0.1 kUA/L, which corresponded to a 100% decrease in their concentration. In the serum of Patient 1, whose baseline anti-beef IgE concentration was several dozen times higher than in Patient 2 or Patient 3 (26 kUA/L vs. 0.38 kUA/L vs. 0.51 kUA/L) after the inhibition test on the beef-coated membrane (f27-IT) anti-beef IgE antibodies were still present, but their concentration decreased by 96.19% (Table 1).

The multiparameter ALEX2 platform showed a 100% reduction in anti-beef IgE antibodies (anti-Bos d_meat IgE) after f-27-IT in all sera, regardless of the initial concentration of these antibodies (Table 1).

The concentrations of IgE antibodies specific to non-beef mammalian meat (sheep, porcine, horse, rabbit and mutton) after inhibition on the f27 coated ImmunoCAP membrane (f27-IT) decreased by less than 6% (Table 1).

Antibodies to poultry and bovine serum albumin (Bos d 6) and porcine serum albumin (Sus d1) remained negative after the f27-ImmunoCAP inhibition test (Table 1).

### 3.4. Control Results

In the ALEX2 test performed on the material collected after the inhibition tests (o215-IT and f27-IT), no characteristic changes in individual sensitization profiles to various airborne and food allergens (other than mammalian meat allergens) were observed compared to the ALEX2 results obtained before the inhibition tests. The results are presented in Table 2.

Changes in the concentration of specific IgE antibodies not exceeding 10% were observed in each patient in both inhibition tests (o215-IT: −6.82% to 5.12% and F27-IT: −6.45% to 3.12%). These changes were multidirectional even for the same allergens. Both a decreases and increases in the concentration of specific IgE for individual allergens after inhibition tests were observed.

## 4. Discussion

Inhibition tests based on immunoblots and ELISA are successfully used to test cross-reactivity between allergens, especially of natural origin. Solid phase inhibition tests based on immunoblots and ELISAs are successfully used to test cross-reactivity between allergens, especially of natural origin. However, they have some limitations resulting from the methodology and low availability of standardized allergen extracts. These types of tests often use self-extracted allergens from natural sources. The composition and stability of these types of extracts are often incomparable. This limits the reproducibility of the results obtained with these methods. Differences in allergen extraction techniques as well as a number of other methodological differences make it difficult to compare the results of ELISA inhibition tests between different research centers. On the other hand, the use of liquid extracts of allergens in inhibition tests (liquid phase inhibition tests) leads to dilution of the test serum and changes in the immune reaction environment, which may affect the final results of the experiment and make their interpretation difficult [6,8,9,10,11,12]. In this manuscript, we proposed an experimental model solid phase two-sided inhibition test on ready-made ImmunoCAP membranes (CAP). We tested the proposed model using the ability of IgE antibodies specific for α-Gal to bind to molecules of this carbohydrate determinant expressed on mammalian meat proteins. This phenomenon underlies the α-Gal syndrome (AGS) [16,17].

In our experiment, we used two types ImmunoCAP membranes (CAP): one coated with α-Gal (bovine thyroglobulin) and the other with beef protein extract. We performed the inhibition tests in sera collected from three patients with different atopic status. We assumed three conditions qualifying the sera for the experiment: the presence of specific anti-α-Gal IgE antibodies, the presence of specific anti-beef IgE antibodies and the absence of poultry-specific IgE antibodies. Allergy to α-Gal results in adverse reactions to mammalian meat, with tolerance to poultry (turkey, chicken) and fish [22]. Poultry proteins and lipids do not show the presence of α-Gal on their surface [23].

To determine anti-α-Gal IgE antibodies, we used two currently available in vitro diagnostic (IVD) tests: the single-component ImmunoCAP method and the multi-component ISAC platform. We found that the results obtained with both methods are consistent only when the serum concentration of anti-α-Gal IgE is high. The higher the concentration of anti-α-Gal IgE antibodies measured by the single-component technique, the higher the concentration of these antibodies in the ISAC assay. Very low levels may not be detected in multi-component techniques. These differences result from the different sensitivity and specificity of the singleplex and multiplex tests used in the routine diagnosis of allergies. They may also be due to the quality and sourcing of the allergens used in the various tests [24].

Antibodies against different mammalian meats were present in the sera of our patients, and the sensitization profile was different. The antibody profile for mammalian meat in the ImmunoCAP assay was not consistent with the antibody profile in the multiparameter ALEX assay. This may be due to methodological differences between these tests and possibly to different sources of allergen extracts used in the two techniques. It is worth noting, however, that antibodies to beef, constituting a selection criterion for us, turned out to be the only antibody to meat allergens present in every patient and detected by every test. Beef sensitization is mentioned by some authors as the main, and sometimes even the only, carnivorous symptom of α-Gal allergy [24,25,26]. Our results seem to be in line with these suggestions. No IgE antibodies specific to poultry allergens were detected in the serum of any of the patients. The antibodies against bovine (Bos d 6) and porcine (Sus d 1) serum albumin, which are specific markers of beef and pork allergy, were also negative [27].

In our study, we observed that bovine allergens abolished the reactivity of patients’ sera to α-Gal by an average of 58%. On the other hand, α-Gal reduced serum reactivity to beef by an average of 91% as assessed by the ImmunoCAP test. In the ALEX2 assay performed with sera blocked to ImmunCAP α-Gal membranes, the inhibition was 100%. Such results seem to confirm that the experimental model of inhibition test on ImmunCAP membranes used by us is effective in the study of cross-reactivity. The differences in the inhibition strength observed between the systems used are probably due to the amount of α-Gal molecules present on the ImmunoCAP membranes. Kollmann et al. [28] observed that α-Gal IgE-positive sera from patients selectively recognize the α-Gal epitope on bovine gamma globulin (BGG) and that incubation of these sera with α-Gal completely blocks this reactivity. The α-Gal epitope is commonly present in bovine IgE-reactive proteins recognized by patients with meat allergy [29,30], and bovine thyroglobulin is considered a very good marker antigen for α-Gal allergy [29,31] and a rich source this carbohydrate determinant [27].

It is noteworthy that the inhibition on the “ImmunoCAP/beef” membrane (f27-IT) was not as effective as the inhibition on the “ImmunoCAP/α-Gal” membrane (o215-IT) for both α-Gal-IgE antibodies and mammalian meat allergens. Binding of anti-α-Gal IgE antibodies to bovine allergens was less efficient than binding to bovine thyroglobulin (α-Gal antigen). However, in the case of reactions specific to allergens of mammalian meat, the reaction with beef allergens was mainly blocked. This result fully meets our expectations and is probably related to the natural distribution of α-Gal glycoprotein in mammalian tissues [27,28,32,33,34,35]. It is worth noting, however, that in the case of our experiment, unambiguous conclusions are hindered by the fact that we do not know the exact composition of the extract that coats the ImmunoCAP bovine membrane. The manufacturer of this membrane only states that these are beef proteins, without specifying their origin [36]. It seems, however, that even the unconventional use of ready-made membranes, which are certified for in vitro diagnostics, should give repeatable results. The extracts that cover the membranes are certainly validated.

It also seems necessary to note that in natural sources, the availability of an antigen for specific antibodies also depends on the relationship between the various molecules present in that material. Many independent factors affect the binding of an antigen to a specific antibody [37,38]. This may be the reason for the different inhibition efficiency using ImmunoCAP membranes coated with an allergen extract (e.g., which we also observed in our studies.

In our experimental inhibition tests, we used ready-made ImmunoCAP membranes, which are the standard in routine in vitro allergy diagnostics. ImmunoCAP allergen membranes have previously been successfully used in inhibition assays for cross-reactivity testing by our team [3,11]. Other researchers also successfully used unconventional experimental methods in the study of cross-reactivity of allergens [23,24,25,29].

Our experience also has a few limitations that we are aware of. The use of natural sera as a source of specific antibodies entails consequences resulting, for example, from the fact that they are mixtures of polyclonal antibodies directed against different epitopes of the same antigen. Different patients were naturally immunized under different conditions, so results obtained with sera from different patients may not be consistent. It is also possible that for this reason the results of such an experiment may not be reproducible. In addition, we used research techniques routinely used in diagnostics, but not in accordance with their intended purpose. Undoubtedly, this could have influenced the obtained results. We also realize that carrying out the experiment on only three sera is not reliable. However, we were unable to obtain more α-Gal positive sera, as allergy to α-Gal in Poland is not a common phenomenon. So far, only two reports on this topic have been published—one population study [39] and one case report [40].

However, these are the results of preliminary studies, which certainly require additional testing using different pairs of cross-reacting allergens with known mutual reactivity. Despite these shortcomings, we believe that the inhibition test model we propose is effective in testing cross-reactivity. We believe that it may find wider application in the assessment of this phenomenon for various allergens. According to our opinion and suggestions of other authors [6], all inhibition tests used to study the phenomenon of cross-reactivity are a valuable tool for observing the relationship between the allergen and the specific antibody in natural conditions.

## 5. Conclusions

In conclusion, the example system of cross-reactive allergens (α-Gal/mammal meat) tested on the proposed experimental model of the two-sided inhibition test on ImmunoCAP membranes allowed to observe that both the beef allergen extract and α-Gal (bovine thyroglobulin), coated on the ImmunoCAP membrane bind anti-α-Gal IgE antibodies present in the serum, which reduces the reactivity of anti-α-Gal IgE sera positive after the inhibition test with mammalian meat allergens, especially beef. These preliminary studies suggest that the experimental model of the two-sided inhibition test on ImmunoCAP membranes proposed by us may be an attractive, simple tool for direct (in real-world) testing of cross-reactivity between allergens. However, because these are preliminary reports from a small sample, they certainly require further research on various allergens with known cross-reactivity.

## 6. Study Limitations and Future Perspectives

Our study has some limitations. First of all, it was carried out only in three patients, which significantly hinders both the analysis of the results and conclusions. This strategy also makes any statistical analysis of the obtained results impossible. The α-Gal syndrome, however, is an extremely rare phenomenon, especially in the area of northern Poland, where the study was conducted. It turned out to be impossible for us to gather a larger study group. In preparing this project, we tested a group of 70 patients, 30 of whom had a confirmed tick bite history, 30 had positive IgE specific antibodies to beef and/or pork allergens and 10 were never bitten by ticks and had no IgE specific positive results. Of this group, only four patients were anti-α-Gal IgE positive, and only three of them were found to have both anti-α-Gal IgE and anti-beef IgE antibodies. We included these three patients in our experimental project, and we performed both inhibition tests (o215-IgE and f27-IgE) in the sera collected from them. Therefore, we are aware that inference based on the results obtained by us may be biased and certainly requires further research on a much larger group of patients. However, this is only a preliminary report on the results of the project, which we will continue with more samples and for more allergens. The concept of the experience seemed interesting and worth presenting at this initial stage.

Despite these limitations, it seems to us that the presented experimental models can be a useful tool for studying the cross-reactivity of various allergens.

## Figures and Tables

**Table 1:** Laboratory test results of the patients (case-by-case), before and after inhibition tests.

Measured Analyte	Inhibition Test (IT)-Stage	Results			p Wilcoxon Test						p Friedman ANOVA	[%] Inhibition		
		Patient 1	Patient 2	Patient 3								Patient 1	Patient 2	Patient 3
ALEX2 Results														
Total IgE [kU/L]	before IT	2602	714	134							0.05			
	after o215-IT	160	20	114	0.11							93.85	97.2	14.93
	after f27-IT	1484	478	128			0.11					42.99	33.1	4.47
Bos d_meat (beef) IgE [kUA/L]	before IT	2.03	3.18	0.53							0.05			
	after IT o215	<0.1	<0.1	<0.1	0.11							100	100	100
	after IT f27	<0.1	<0.1	<0.1			0.11					100	100	100
Sus d_meat (pork) IgE [kUA/L]	before IT	0.38	0.49	<0.1							0.13			
	after o215-IT	0.21	<0.1	<0.1	0.18							44.74	100	-
	after f27-IT	0.36	0.46	<0.1			0.18					5.26	6.12	-
Equ c_meat (horseflesh) IgE [kUA/L]	before IT	1.8	3.17	0.25							0.05			
	after o215-IT	<0.1	0.11	0.2	0.11							100	96.53	20
	after f27-IT	1.7	3.12	0.25			0.18					5.5	1.58	0
Ovi a_meat (lamb/mutton) IgE [kUA/L]	before IT	<0.1	0.59	<0.1							0.36			
	after o215-IT	<0.1	<0.1	<0.1	-							-	100	-
	after f27-IT	<0.1	0.58	<0.1			-					-	1.69	-
Oryc_meat (rabbit meat) IgE [kUA/L]	before IT	0.76	0.53	<0.1							0.22			
	after o215-IT	<0.1	<0.1	<0.1	0.18							100	100	-
	after f27-IT	0.45	0.53	<0.1			0.65					1.3	0	-
Gal d_meat (chicken) IgE [kUA/L]	before IT	<0.1	<0.1	<0.1							-			
	after o215-IT	<0.1	<0.1	<0.1	-							-	-	-
	after f27-IT	<0.1	<0.1	<0.1			-					-	-	-
Mel d_meat (turkey) IgE [kUA/L]	before IT	<0.1	<0.1	<0.1							-			
	after o215-IT	<0.1	<0.1	<0.1	-							-	-	-
	after f27-IT	<0.1	<0.1	<0.1			-					-	-	-
Bos d 6 IgE [kUA/L] (serum albumin)	before IT	<0.1	<0.1	<0.1							-			
	after o215-IT	<0.1	<0.1	<0.1	-							-	-	-
	after f27-IT	<0.1	<0.1	<0.1			-					-	-	-
Sus d 1 IgE [kUA/L] (serum albumin)	before IT	<0.1	<0.1	<0.1							-			
	after o215-IT	<0.1	<0.1	<0.1	-							-	-	-
	after f27-IT	<0.1	<0.1	<0.1			-					-	-	-
ISAC*_E112i_* results														
anti-α-Gal IgE (nGal-alpha-1,3-Gal) [ISU-E]	before IT	12	0.3	<0.3										
ImmunoCAP results														
anti-α-Gal IgE (nGal-alpha-1,3-Gal) [kUA/L]	before IT	302	35.8	0.7							0.049			
	after o215-IT	43.6	4.28	<0.1	0.11							85.56	88.04	100
	after f27-IT	161	26.1	<0.1					0.11			46.69	27.09	100
anti-f27 (beef) IgE [kUA/L]	before IT	26	0.38	0.51							0.059			
	after o215-IT	16.76	0.21	<0.1	0.11							35.54	44.74	100
	after f27-IT	0.99	<0.1	<0.1					0.11			96.19	100	100
anti-f26 (pork) IgE [kUA/L]	before IT	7.2	<0.1	<0.1										
anti-f213 (rabbit meat) [kUA/L] IgE	before IT	1.64	<0.1	<0.1										
anti-f88 (mutton) IgE [kUA/L]	before IT	2.47	<0.1	<0.1										
anti-c74 (gelatine) IgE [kUA/L]	before IT	<0.1	<0.1	<0.1										
anti-f83 (chicken) IgE [kUA/L]	before IT	<0.1	<0.1	<0.1										

**Legend:** IT—inhibition test; o215-IT—ImmunoCAP inhibition test with α-Gal; f27-IT—ImmunoCAP inhibition test with beef; p—statistical significance factor; statistically significant results (*p* ≤ 0.05) are in bold; Wilcoxon test: after o215-IT vs. before IT; after f27-IT vs. before IT; Friedman ANOVA: before IT vs. o215-IT vs. after f27-IT.

**Table 2:** Concentration and percentage changes in the concentration of specific IgE for allergens other than mammalian meat, poultry meat and α-Galibefore the inhibition tests on the ImmunoCAP o215 matrix (o215-IT) and the ImmunoCAP f27 matrix (f27-IT) and after the inhibition tests (case-by-case).

Control Analyte	Inhibition Test (IT)-Stage	Result	[%] Inhibition
Patient 1			
Aln g 1 IgE (alder pollen; PR-10) [kU/L]	before IT	0.49	
	after o215-IT	0.51	−4.08
	after f27-IT	0.48	2.04
Amb a 1 IgE (ragweed pollen; pectate lyase) [kUA/L]	before IT	0.40	
	after IT o215	0.41	−2.5
	after IT f27	0.41	−2.5
Gly m 5 IgE (soya bean; 7S vicilin) [kUA/L]	before IT	1.24	
	after o215-IT	1.25	−0.8
	after f27-IT	1.32	−6.45
Act d 1 IgE (kiwi fruit; actinidin) [kUA/L]	before IT	0.62	
	after o215-IT	0.65	−4.83
	after f27-IT	0.64	−3.22
Mal d 1 IgE (apple, PR-10) [kUA/L]	before IT	0.44	
	after o215-IT	0.47	−6.82
	after f27-IT	0.44	0
Ves v 5 IgE (wasp venom, antigen 5) [kUA/L]	before IT	1.31	
	after o215-IT	1.32	−0.76
	after f27-IT	1.30	0.76
Patient 2			
Bet v 1 IgE (birch pollen, PR-10) [kU/L]	before IT	0.63	
	after o215-IT	0.62	1.59
	after f27-IT	0.64	−1.59
Ara h 8 IgE (peanut; PR-10) [kUA/L]	before IT	0.33	
	after IT o215	0.35	−6.06
	after IT f27	0.32	3.12
Act d 2 IgE (kiwi fruit; thaumatin-like protein) [kUA/L]	before IT	0.48	
	after o215-IT	0.48	0
	after f27-IT	0.51	−6.25
Canf 4 IgE (domestic dog; lipocalin) [kUA/L]	before IT	1.79	
	after o215-IT	1.83	−2.23
	after f27-IT	1.80	−0.56
Fel d 4 IgE (domestic cat; lipocalin) [kUA/L]	before IT	0.35	
	after o215-IT	0.36	−2.86
	after f27-IT	0.37	−5.71
Patient 3			
Aln g 1 (alder pollen; PR-10) [kU/L]	before IT	3.93	
	after o215-IT	3.98	−1.27
	after f27-IT	3.9	0.76
Bet v 1 IgE (birch pollen, PR-10) [kUA/L]	before IT	30.49	
	after IT o215	29.46	3.38
	after IT f27	30.51	−0.07
Cor a 1.0103 IgE (hazel pollen, PR-10) [kUA/L]	before IT	4.52	
	after o215-IT	4.58	−1.33
	after f27-IT	4.55	−0.66
Amb a 4 IgE (ragweed pollen; defensin-like protein) [kUA/L]	before IT	1.79	
	after o215-IT	1.89	−5.59
	after f27-IT	1.89	−5.59
Art v 1 IgE (mugwort pollen; defensin-like protein) [kUA/L]	before IT	17.85	
	after o215-IT	17.85	0
	after f27-IT	17.82	0.17
Ara h 8 IgE (peanut; PR-10) [kUA/L]	before IT	1.68	
	after o215-IT	1.65	1.79
	after f27-IT	1.67	0.6
Gly m 4 IgE (soya bean; PR-10) [kUA/L]	before IT	2.01	
	after IT o215	1.98	1.49
	after IT f27	1.99	1
Api g 1 IgE (celery root; PR-10) [kUA/L]	before IT	3.86	
	after o215-IT	3.84	0.52
	after f27-IT	3.87	−0.26
Cor a 1.0401 IgE (hazelnut; PR-10) [kUA/L]	before IT	6.62	
	after o215-IT	6.62	0
	after f27-IT	6.64	−0.3
Ani s 3 IgE (Anisakis simplex; tropomyosin) [kUA/L]	before IT	0.31	
	after o215-IT	0.31	0
	after f27-IT	0.32	−3.23
Can f urine (Can f 5) IgE (domestic dog; arginine esterase) [kUA/L]	before IT	3.99	
	after o215-IT	4	−0.25
	after f27-IT	4.01	−5
Can f 1 IgE (domestic dog; lipocalin) [kUA/L]	before IT	25.5	
	after IT o215	24.8	2.75
	after IT f27	24.81	2.71
Fel d 7 IgE (domestic cat; lipocalin) [kUA/L]	before IT	6.49	
	after o215-IT	6.73	−3.7
	after f27-IT	6.69	−3.1
Ves v 5 IgE (wasp venom, antigen 5) [kUA/L]	before IT	0.39	
	after IT o215	0.37	5.12
	after IT f27	0.4	−2.56

**Legend:** IT—inhibition test; o215-IT—ImmunoCAP inhibition test with α-Gal; f27-IT—ImmunoCAP inhibition test with beef; [%] inhibition—negative values indicate an increase in concentration after the inhibition test, and positive values a decrease in concentration after the inhibition test; PR-10: Pathogenesis-Related protein 10.

## Data Availability

Not applicable.
